# The clinicopathological significance of the adipophilin and fatty acid synthase expression in salivary duct carcinoma

**DOI:** 10.1007/s00428-020-02777-w

**Published:** 2020-02-26

**Authors:** Hideaki Hirai, Yuichiro Tada, Masato Nakaguro, Daisuke Kawakita, Yukiko Sato, Tomotaka Shimura, Kiyoaki Tsukahara, Satoshi Kano, Hiroyuki Ozawa, Kenji Okami, Yuichiro Sato, Chihiro Fushimi, Akira Shimizu, Isaku Okamoto, Soichiro Takase, Takuro Okada, Hiroki Sato, Yorihisa Imanishi, Kuninori Otsuka, Yoshihiro Watanabe, Akihiro Sakai, Koji Ebisumoto, Takafumi Togashi, Yushi Ueki, Hisayuki Ota, Natsuki Saigusa, Hideaki Takahashi, Mizuo Ando, Makoto Urano, Toyoyuki Hanazawa, Toshitaka Nagao

**Affiliations:** 1 Department of Anatomic Pathology, Tokyo Medical University, 6-7-1 Shinjuku, Shinjuku-ku, Tokyo, 160-0023 Japan; 2 Department of Head and Neck Oncology and Surgery, International University of Health and Welfare Mita Hospital, Tokyo, Japan; 3 Department of Pathology and Laboratory Medicine, Nagoya University Hospital, Nagoya, Japan; 4 Department of Otorhinolaryngology, Head and Neck Surgery, Nagoya City University Graduate School of Medical Sciences, Nagoya, Japan; 5 Division of Pathology, Cancer Institute Hospital, Japanese Foundation for Cancer Research, Tokyo, Japan; 6 Department of Otolaryngology, Showa University Fujigaoka Hospital, Kanagawa, Japan; 7 Department of Otolaryngology, Head and Neck Surgery, Tokyo Medical University, Tokyo, Japan; 8 Department of Otolaryngology - Head and Neck Surgery, Faculty of Medicine and Graduate School of Medicine, Hokkaido University, Sapporo, Japan; 9 Department of Otorhinolaryngology, Head and Neck Surgery, Keio University School of Medicine, Tokyo, Japan; 10 Department of Otolaryngology, School of Medicine, Tokai University, Kanagawa, Japan; 11 Department of Head and Neck Surgery, Niigata Cancer Center Hospital, Niigata, Japan; 12 Department of Otorhinolaryngology, Head and Neck Surgery, School of Medicine, Yokohama City University, Yokohama, Japan; 13 Department of Otolaryngology - Head and Neck Surgery, Faculty of Medicine, The University of Tokyo, Tokyo, Japan; 14 Department of Diagnostic Pathology, School of Medicine, Fujita Health University, Toyoake, Japan; 15 Department of Otorhinolaryngology/Head & Neck Surgery, Chiba University Graduate School of Medicine, Chiba, Japan

**Keywords:** Salivary duct carcinoma, Adipophilin, Fatty acid synthase, Lipid metabolism, Prognosis, Androgen receptor

## Abstract

Salivary duct carcinoma (SDC) is an aggressive, uncommon tumor histologically comparable to high-grade mammary ductal carcinoma. SDCs are usually androgen receptor (AR)–positive and often HER2-positive. Recently, therapies targeting these molecules for SDC have attracted attention. Lipid metabolism changes have been described in association with biological behavior in various cancers, although no such relationship has yet been reported for SDC. We therefore analyzed the clinicopathological relevance of the immunohistochemical expression of adipophilin (ADP) and fatty acid synthase (FASN), representative lipid metabolism–related proteins, in 147 SDCs. ADP and FASN were variably immunoreactive in most SDCs (both 99.3%), and the ADP and FASN expression was negatively correlated (*P =* 0.014). ADP-positive (≥ 5%) SDCs more frequently exhibited a prominent nuclear pleomorphism and high-Ki-67 labeling index than those ADP-negative (*P =* 0.013 and 0.011, respectively). In contrast, a high FASN score, calculated by the staining proportion and intensity, (≥ 120) was correlated with the high expression of AR and FOXA1 (*P* < 0.001 and = 0.003, respectively). The ADP and FASN expression differed significantly among the subtypes based on biomarker immunoprofiling, as assessed by the AR, HER2, and Ki-67 status (*P* = 0.017 and 0.003, respectively). A multivariate analysis showed that ADP-positive expression was associated with a shorter overall and progression-free survival (*P =* 0.018 and 0.003, respectively). ADP was associated with an aggressive histopathology and unfavorable prognosis, and FASN may biologically interact with the AR signaling pathway in SDC. ADP may, therefore, be a new prognostic indicator and therapeutic target in SDC.

**Electronic supplementary material:**

The online version of this article (10.1007/s00428-020-02777-w) contains supplementary material, which is available to authorized users.

## Introduction

Salivary duct carcinoma (SDC) is an aggressive and uncommon tumor that accounts for as many as 10% of all salivary gland malignancies. It can occur not only as de novo carcinoma but also as a malignant component of carcinoma ex pleomorphic adenoma [1]. SDC is histologically comparable to high-grade mammary ductal carcinoma and recent transcriptome data have also shown striking similarities between SDC and apocrine breast cancer [2]. Most SDCs express androgen receptor (AR) and its related protein, FOXA1, but not estrogen receptor (ER) or progesterone receptor, and approximately 40% are positive for HER2 [3, 4].

While normal cells produce energy by aerobic phosphorylation through the tricarboxylic acid cycle, cancer cells produce energy by aerobic glycolysis and other metabolic pathways. The oncogenic metabolic pathways differ depending on the tumor types and, therefore, the development of therapies against tumor metabolism is not straightforward [5]. Lipid metabolism is a crucial pathway in tumor progression and cancer cells typically show lipid accumulation [6, 7].

Adipophilin (ADP), also known as perilipin 2 or adipose differentiation–related protein, covers intracytoplasmic lipid droplets. ADP prevents the efflux of intracellular lipid droplets and increases the intracellular lipid level [8]. Immunohistochemically, ADP is a diagnostic marker of apocrine differentiation in breast cancer, and lipid-storage cancers such as sebaceous carcinoma and secretory carcinoma of the salivary glands [9–12]. Furthermore, the ADP expression was recently found to be a poor prognosticator in various malignant tumors, including pulmonary, pancreatic, colonic, and renal cell carcinomas, and Burkitt lymphoma [13–17]. In breast cancer, ADP is related to the ER expression, HER2 status, and the classification based on the biomarker immunoprofiling [9].

Fatty acid synthase (FASN) is a key lipogenic enzyme and is also overexpressed in various human cancers including salivary tumors [18, 19]. FASN expression has been reported to be associated with a poor prognosis in several types of tumors [20, 21]. In addition, in breast cancer, like ADP, the FASN expression is correlated with the HER2 status and the subtype of the immunoprofiling classification, and in prostate cancer, the FASN expression is correlated with the AR expression [22–24].

To our knowledge, the role of lipid metabolism–related proteins in SDCs has not yet been described. In the present study, we immunohistochemically evaluated the ADP and FASN expression and investigated their clinicopathological significance in a series of 147 SDCs.

## Materials and methods

This study was approved by the Institutional Ethics Review Board of each participating institution.

### Patients

All patients underwent a central pathological review by an expert pathologist (T.N.) according to the rigorous histomorphologic criteria for SDC. We recruited 147 patients who were diagnosed with and received treatment for SDC at seven institutions between 1992 and 2014. Patients who were treated with anti-AR or anti-HER2 therapy as a first-line treatment were excluded from this study. We retrospectively reviewed the patient records to obtain information about the age, sex, tumor size, lymph node metastasis, other distant metastasis, and outcome. Tumor staging was classified in accordance with the seventh ed. TNM classification of the International Union Against Cancer 2009.

### Histopathology

The histopathological analysis of nuclear pleomorphism was based on our previous report [25]. In brief, we evaluated the presence of marked variation in the size of tumor cell nuclei throughout the tumor, rather than focal, regardless of the absolute nuclear size.

### Immunohistochemistry (IHC) and fluorescence in situ hybridization (FISH)

For IHC, formalin-fixed, paraffin-embedded tumor tissue was cut into 4-μm-thick sections. A polymer-based detection system with heat-mediated antigen retrieval was conducted using the primary antibodies shown in Supplementary Table 1. Diaminobenzidine was applied to detect antigen-antibody reactions. The ADP expression was considered to be positive when the globular or granular cytoplasmic expression was found in ≥ 5% of the tumor cells, as previously reported [13, 15]. FASN was analyzed using a combined scoring system based on both the proportion of positive tumor cells (0–100%) and the predominant staining intensity in the tumor [26]. The FASN staining intensity was scored categorically (0, negative; 1, weak; 2, moderate; 3, strong). FASN score (0–300) was calculated by multiplying the percentage by the staining intensity. SDC cases were classified into two groups based on FASN score: low (< 120) and high (≥ 120). A case was considered high for AR and FOXA1 when ≥ 20% of the tumor cell nuclei showed strong staining [4, 27]. HER2 was considered to be positive based on an HER2 IHC score of 3+ and/or *HER2* amplification, as determined by a FISH analysis, in accordance with the ASCO/CAP guideline for evaluating breast cancer [28]. The results of p53 staining were interpreted based on the expression pattern. Cases were classified into three groups as follows: extreme negative, complete confluent negativity of staining; extreme positive, strong diffuse confluent positivity; and non-extreme, all intermediate expression of any intensity [27]. The percentage of Ki-67-positive cells was determined by counting at least 1000 tumor cells, and then recorded as the Ki-67 labeling index (LI). Ki-67 LI values of < 40% and ≥ 40% were classified as Ki-67-low and Ki-67-high, respectively [9]. The HER2 status and the results of immunohistochemical staining of AR, FOXA1, Ki-67, and p53, which were previously reported by our group, were compared to the ADP and FASN expression [4, 27, 29].

### Classification based on the biomarker immunoprofile

The classifications of SDC were based on our previous description. All SDCs were categorized into five main subtypes based on a combination of the expression of AR (instead of ER or PR for breast cancer), HER2 (or the *HER2* amplification status), and Ki-67 as follows: “apocrine A” (AR+/HER2−/Ki-67-low), “apocrine B” (AR+/HER2−/Ki-67-high), “apocrine HER2” (AR+/HER2+), “HER2-enriched” (AR−/HER2+), and “double-negative” (AR−/HER2−) [27].

### Statistical analyses

Non-continuous variables were compared using the chi-squared test. Continuous variables were compared using the Mann-Whitney *U* test. Spearman’s rank correlation test was used to evaluate the association between the protein expression. The association between the ADP expression and overall survival (OS) or progression-free survival (PFS) was evaluated using the Kaplan-Meier product-limit method and univariate and multivariate Cox proportional hazard models. The potential confounders in the multivariate analysis included the age (< 65 vs. ≥ 65 years), sex (male vs. female), primary tumor site (parotid gland vs. submandibular gland vs. other), T classification (1–4), N classification (0 vs. 1, 2), M classification (0, 1), first-line treatment (surgery vs. other), and histological type (de novo vs. carcinoma ex pleomorphic adenoma). All statistical tests were performed using the STATA software program (version 13, StataCorp, College Station, TX, USA). All tests were two-sided, and *P* values of < 0.05 were considered to indicate statistical significance.

## Results

### Histological features and patient characteristics

**Fig. 1:**
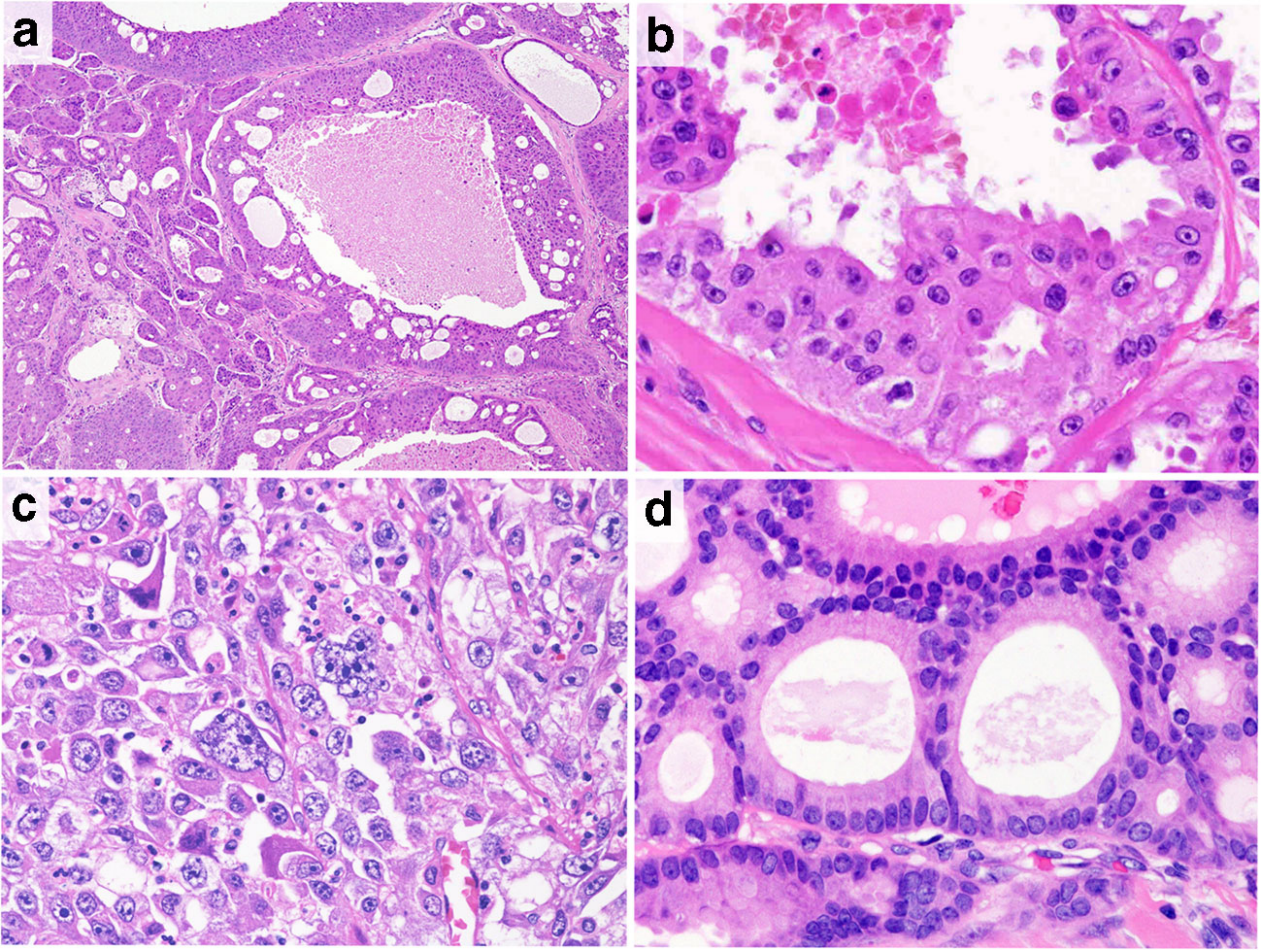
Representative histologic features of salivary duct carcinoma (SDC). **a** SDC consisted of intraductal and invasive components. **b** Carcinoma cells have abundant, granular, and eosinophilic cytoplasm and display an apocrine snout-like morphology (i.e.*,* apocrine-like features). **c** An example of a SDC case with prominent nuclear pleomorphism. **d** An example of a SDC case without prominent nuclear pleomorphism

Representative histological findings in a case of SDC are shown in Fig. 1. All cases of SDC had abundant granular and eosinophilic cytoplasm and sometimes displayed an apocrine snout-like morphology (i.e.*,* apocrine-like features). Histopathological variants of SDC were identified as sarcomatoid (*n* = 9; 6.1%), invasive micropapillary (*n* = 6; 4.1%), and mucin-rich (*n* = 2; 1.4%) variants. The patient characteristics are shown in Table 1. Prominent nuclear pleomorphism was observed in 97 cases (66%) (Figs. 1c, d). The median follow-up period of the survivors was 3.4 years (range, 0.04–19.0 years). The 3-year OS rate in all patients was 69.0% (95% confidence interval [CI], 60.5%–76.0%), while the 3-year PFS rate was 37.5% (95% CI, 29.4%–45.6%).

**Table 1:** Patient characteristics and the correlation between the adipophilin/FASN expression and clinicopathological factors

Clinicopathological factors	*n* (%)	Adipophilin expression		*P*	FASN score		*P*
		negative (< 5%)	Positive (≥ 5%)		Low (< 120)	High (≥ 120)	
		*n* = 35	*n* = 112		*n* = 30	*n* = 117	
FASN							
Low	30 (20)	2	28	0.014*			
High	117 (80)	33	84				
Age, mean ± SD, years		62.0 ± 13.0	61.9 ± 12.3	0.961	61.7 ± 12.7	62.0 ± 12.4	0.894
Sex							
Male	123 (84)	27	96	0.231	22	101	0.086
Female	24 (16)	8	16		8	16	
T classification							
1–2	51 (35)	16	35	0.117	9	42	0.545
3–4	96 (65)	19	77		21	75	
N classification							
0	70 (48)	20	50	0.196	16	54	0.482
1–2	77 (52)	15	62		14	63	
M classification							
0	138 (94)	35	103	0.084	27	111	0.321
1	9 (6)	0	9		3	6	
Histologic origin							
De novo	56 (39)	17	39	0.203	8	48	0.143
CXPA	86 (61)	18	68		21	65	
Prominent nuclear pleomorphism							
Absent	50 (34)	18	32	0.013*	8	42	0.341
Present	97 (66)	17	80		22	75	
AR							
Negative	31 (21)	4	27	0.109	14	17	<0.001*
Positive	116 (79)	31	85		16	99	
FOXA1							
Negative	20 (14)	2	18	0.108	9	11	0.003*
Positive	120 (86)	32	88		20	100	
HER2 status							
Negative	78 (53)	23	55	0.058	13	65	0.214
Positive	68 (47)	11	57		17	51	
Ki67 LI mean ± SD (%)		35.4 ± 22.4	47.2 ± 23.8	0.011*	49.2 ± 24.2	43.3 ± 23.8	0.231
p53							
NE	83 (57)	24	59	0.072	17	66	0.867
EN/EP	62 (43)	10	52		12	50	

*Abbreviations*: *FASN* fatty acid synthase, *SD* standard deviation, *CXPA* carcinoma ex-pleomorphic adenoma, *AR* androgen receptor, *FOXA1* forkhead box protein A1, *HER2* human epidermal growth factor receptor type 2, *LI* labeling index, *NE* not extreme, *EN/EP* extreme negative/positive

*Statistically significant association (*P* < 0.05)

### The expression of ADP and FASN

**Fig. 2:**
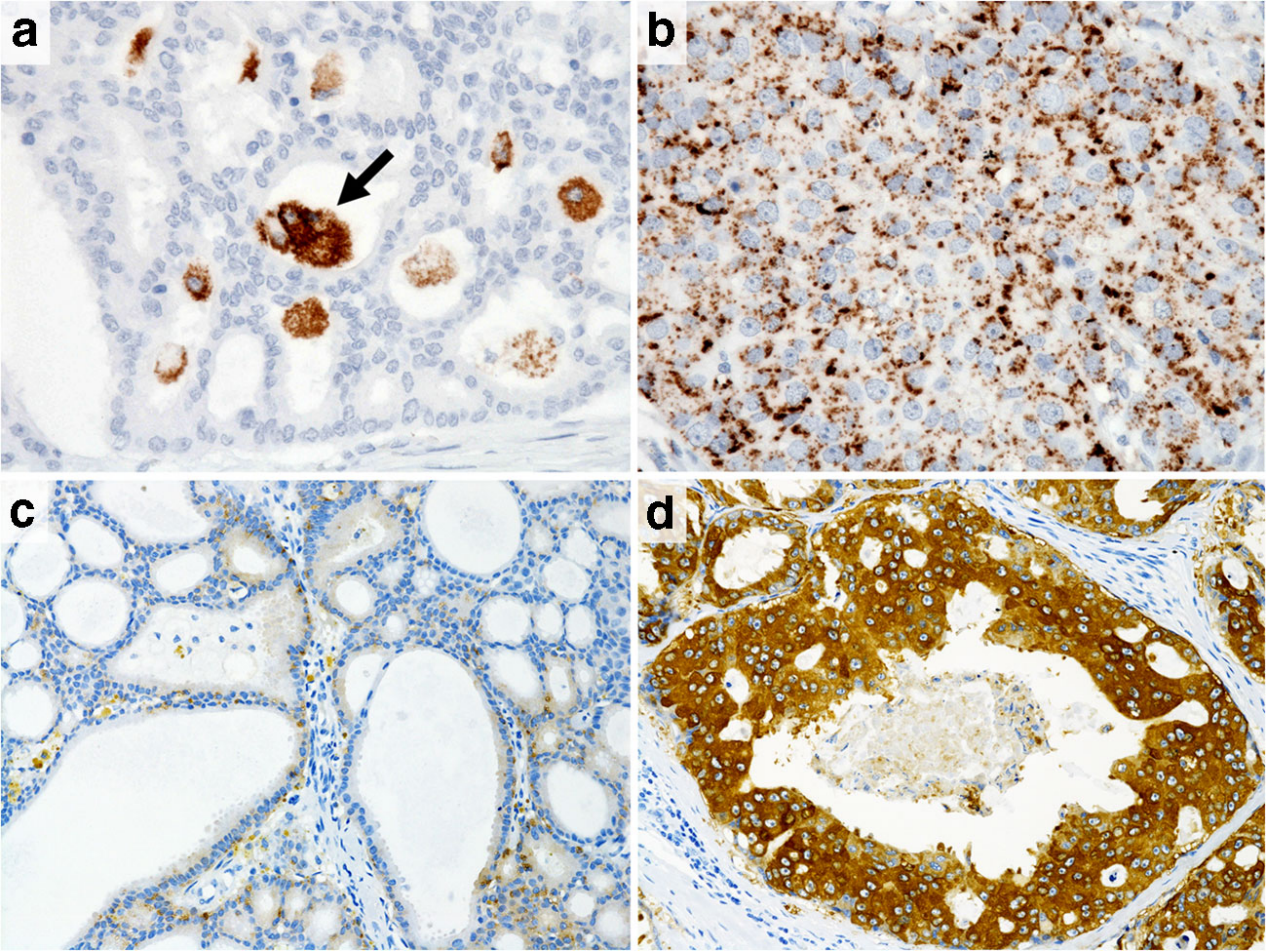
The adipophilin expression is immunohistochemically detected in **a** 0% and, **b** 100% of salivary duct carcinomas. Note that foamy macrophages in the tubular lumen were used as a built-in positive control (arrow) (**a**). The fatty acid synthase score is immunohistochemically **c** low (80) and **d** high (300) in salivary duct carcinomas

Among the 147 SDC tumor specimens, ADP and FASN were expressed in at least a limited part of the tumor in 146 cases (both in 99.3%). The ADP expression of foamy macrophages in the tubular lumen (Fig. 2a) and the FASN expression of the adipose tissue were used as an internal positive control [9, 10]. Thirty-five cases (23.8%) and 112 cases (76.2%) were classified into the ADP-negative and ADP-positive groups, respectively (mean ADP expression value 13.5%) (Figs. 2a, b), and 30 cases (20.6%) and 116 cases (79.4%) were categorized into the FASN-low and FASN-high groups, respectively (mean FASN score 168.1) (Figs. 2c, d).

The correlations between ADP/FASN expression and the clinicopathological factors, and various biomarkers are summarized in Table 1. A negative correlation was observed between the expression of ADP and FASN (*P =* 0.014). An ADP-positive tumor was associated with the presence of a prominent nuclear pleomorphism and a high Ki-67 labeling index (*P =* 0.013 and 0.011, respectively). A marginally significant association was detected between ADP and HER2 positivity (*P =* 0.058). A FASN-high status was associated with high AR and FOXA1 expression levels (*P* < 0.001, and 0.003, respectively). A Spearman’s rank correlation test revealed that the FASN score was correlated significantly and positively with the AR and FOXA1 expression (*r* = 0.315, *P* < 0.001 and *r* = 0.277, *P* < 0.001, respectively). No variables were correlated with sex, age, TNM classification, or histological origin. The ADP and FASN expression levels differed significantly among the different biomarker immunoprofile subtypes (*P* = 0.017 and 0.003, respectively) (Supplementary Table 2). The ratio of cases with ADP-positive was highest in the “HER2-enriched” group and lowest in the “Apocrine A” group, whereas the ratio of cases with a high-FASN expression was highest in the “Apocrine B” group and lowest in the “HER2-enriched” group.

### Prognostic impact

**Fig. 3:**
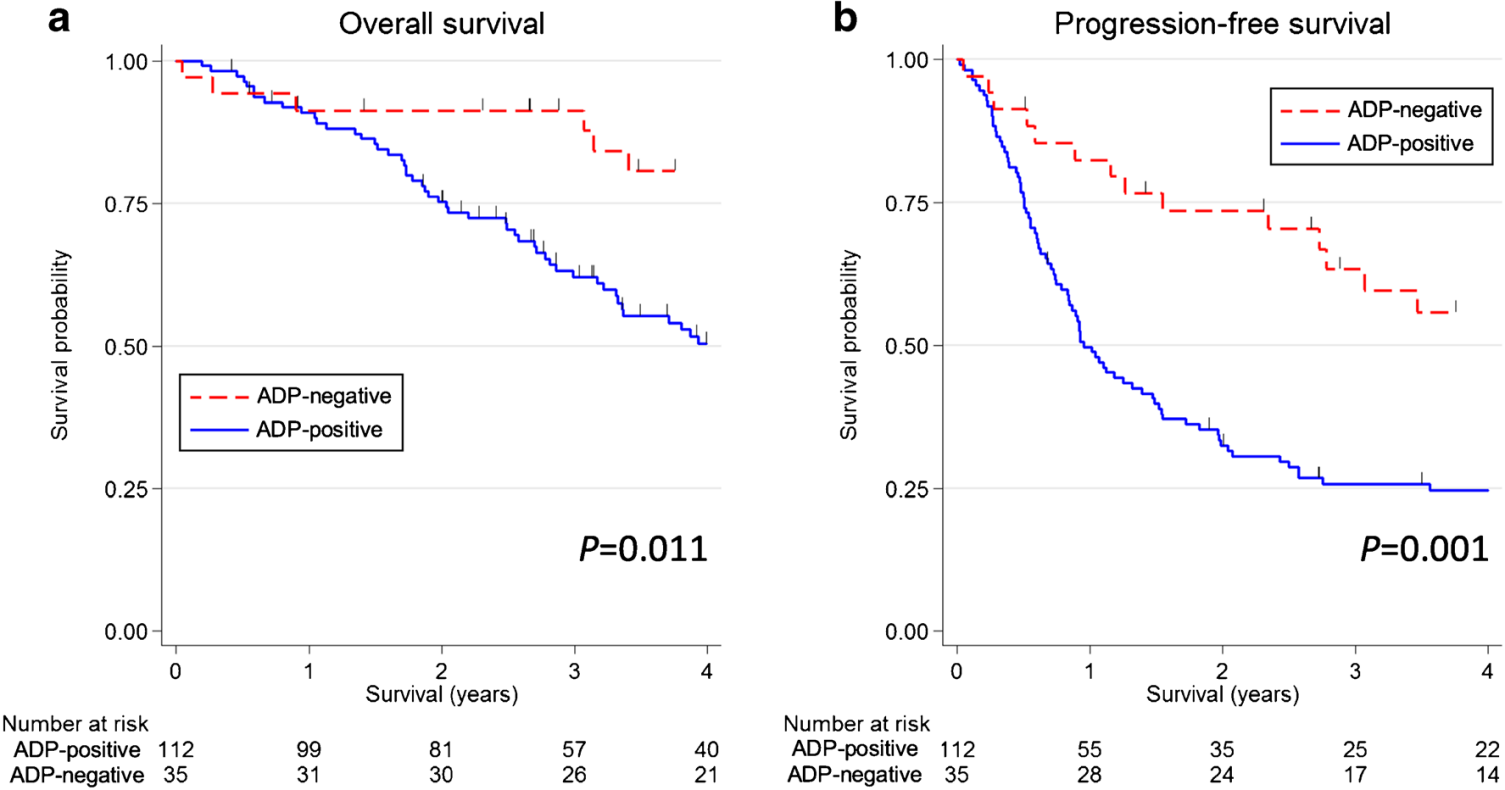
Kaplan-Meier survival curves according to the adipophilin (ADP) expression in salivary duct carcinoma. **a** Patients with ADP-positive show significantly shorter overall survival (*P* = 0.011) and **b** progression-free survival (*P* = 0.001) in comparison to those with ADP-negative

The results of the univariate and multivariate analyses are shown in Table 2. The univariate and multivariate analyses revealed that ADP-positive expression was significantly associated with shorter OS and PFS. The Kaplan-Meier survival curves of the association between the ADP expression and the clinical outcomes are shown in Fig. 3. However, a significant association between the FASN expression and the PFS was found only in the multivariate analysis, not in the univariate analysis. There was no significant association between the FASN expression and the OS.

**Table 2:** Results of the univariate and multivariate analyses of the clinical outcomes in patients with salivary duct carcinoma

	*n*	Overall survival						Progression-free survival					
		Univariate analysis			Multivariate analysis			Univariate analysis			Multivariate analysis		
		HR	95% CI	*P*	HR	95% CI	*P*	HR	95% CI	*P*	HR	95% CI	*P*
Adipophilin expression													
Positive (≥ 5%)	112	1.00	–	–	1.00	–	–	1.00	–	–	1.00	–	–
Negative (< 5%)	35	0.44	0.23–0.84	0.013*	0.43	0.21-0.86	0.018*	0.42	0.25-0.71	0.001*	0.42	0.24-0.74	0.003*
FASN score													
High (≥ 120)	117	1.00	–	–	1.00	–	–	1.00	–	–	1.00	–	–
Low (< 120)	30	1.15	0.65–2.03	0.638	1.25	0.67–2.34	0.484	1.27	0.79–2.05	0.330	1.88	1.12–3.15	0.017*

Adjusted by age, sex, primary tumor site, TNM classification, first-line treatment, and histologic origin

*Abbreviations*: *HR* hazard ratio, *CI* confidence interval, *FASN* fatty acid synthase

*Statistically significant association (*P* < 0.05)

## Discussion

In the present study, we analyzed the lipid metabolism–related protein expression, focusing on ADP and FASN, for their clinicopathological relevance in SDC. We demonstrated that almost all of the 147 SDC cases expressed ADP and FASN, with a diverse ratio and intensity of positive cells, and that there was an association between ADP and FASN expression. ADP-positive expression was associated with the presence of a prominent nuclear pleomorphism (i.e., histological aggressiveness), high Ki-67 labeling index, and a poor prognosis. Furthermore, the high expression of FASN was positively correlated with AR and FOXA1 expression. The ADP and FASN status was also linked with the biomarker-based classification of SDC.

To our knowledge, this is the first study to validate ADP as an independent unfavorable prognostic factor in a large cohort of SDC patients. The following three hypotheses might support the results. First, the high expression of ADP is associated with histological aggressiveness and consequently results in a poor prognosis. Lung and pancreatic cancers with high ADP expression often show high-grade morphological features and poor prognosis [13, 15]. We found a similar relationship in SDC. Second, in SDC, intracellular lipids may stimulate tumor cell proliferation and this could result in an unfavorable prognosis. In a colon carcinoma cell line, the accumulated lipid droplets promoted cellular proliferation, whereas the silencing of ADP inhibited cellular proliferation [30]. In this study, ADP-positive expression was related to a high Ki-67 labeling index, a representative marker of cellular proliferation. This relationship was also reported in lung and breast cancer [9, 13]. Third, in SDC, the expression of ADP may reflect the activation of hypoxic signaling, and this activation may lead to an aggressive phenotype and a poor prognosis. The accumulation of intracellular lipid droplets is a usual observation in ischemic tissues, such as organ infarction [7]. In cancer, hypoxic signaling usually is activated by ischemia and is related to an aggressive phenotype [7, 31]. It is suggested that the coordination of the metabolic deregulation and hypoxic signaling contributed to the biological aggressiveness of lung adenocarcinoma [31]. To verify these three hypotheses, further studies of the functional role of ADP in SDC is required.

In SDC, the FASN score was correlated significantly and positively with the AR and FOXA1 expression. In prostate cancer, selective FASN inhibition antagonizes tumor growth through metabolic reprogramming and results in a reduced protein expression and reduced transcriptional activity of AR. Activation of the endoplasmic reticulum stress response, resulting in reduced protein synthesis, was involved in the FASN inhibition of the AR pathway [24]. In SDC, as well as prostate cancer, the expression of FASN might be associated with the expression of AR through the endoplasmic reticulum stress response. The association between the expression of FASN and FOXA1 in cancer has not been examined. In SDC, the FOXA1 expression was positively correlated with the expression of AR [4]; thus, there might be a positive relationship between FASN and FOXA1. Recently, therapies targeting AR have been shown to be effective in SDC patients and have attracted attention [32]. The overexpression of FASN was reported to be linked to castration-resistant prostate cancer growth and resistance to chemotherapy [24, 33]. Future studies are expected to clarify the association between these therapies and the FASN expression in SDC.

In this study, a negative correlation was observed between the expression of ADP and FASN in SDC. Both positive and negative correlations have been reported in previous studies in different types of cells [34, 35]. ADP reflects the presence of intracellular lipid accumulation well [9, 36]. FASN is known to be a central enzyme in de novo lipogenesis primarily from carbohydrate sources, inducing lipid accumulation [37]. However, lipid accumulation in cells is derived from not only de novo lipogenesis but also the uptake of lipids and neutral lipid synthesis, irrespective of FASN [35]. Further studies will be necessary to clarify these mechanisms and their significance in SDC.

In breast cancer, ADP and FASN have been reported to be associated with the immune-biomarker classification, which is a surrogate for molecular subtyping reflecting the different metabolic pathways [9, 22]. Given that FASN inhibitors have shown cytotoxicity in various cancers [18, 38], the different expressions of ADP and FASN in accordance with the SDC subtypes assessed by the AR (instead of ER or PR for breast cancer), HER2, and Ki-67 status might imply that inhibiting proteins related to lipid metabolism is a possible therapeutic approach for some subtypes. Although we failed to prove the influence of the FASN expression in SDC on the prognosis, patients with a high FASN expression score may be suitable for FASN inhibitor therapy in the future, regardless of the prognostic significance. However, further analyses are needed to establish whether or not lipid metabolism–related proteins are suitable therapeutic targets in SDC.

In conclusion, this study showed that ADP and FASN are frequently but unevenly expressed in SDC. ADP is related to histological aggressiveness and has prognostic significance with an unfavorable survival outcome in SDC patients. ADP may, therefore, be a new prognostic indicator and a novel therapeutic target associated with lipid metabolism. In addition, FASN might biologically interact with the AR signaling pathway.

## Electronic supplementary material

ESM 1 (XLSX 15 kb)
